# Selective modulation of neuronal firing by pulse stimulations with different frequencies in rat hippocampus

**DOI:** 10.1186/s12938-019-0700-z

**Published:** 2019-07-23

**Authors:** Chen Qiu, Zhouyan Feng, Lvpiao Zheng, Weijian Ma

**Affiliations:** Key Laboratory of Biomedical Engineering of Education Ministry, College of Biomedical Engineering and Instrumentation Science, Zhejiang University, Hangzhou, 310027 Zhejiang China

**Keywords:** Electrical pulse stimulation, Hippocampal CA1 region, Interneurons, Pyramidal cells, Unit spike, Stimulation frequency, Silent period

## Abstract

**Background:**

Deep brain stimulation (DBS) has a good prospect for treating many brain diseases. Recent studies have shown that axonal activation induced by pulse stimulations may play an important role in DBS therapies through wide projections of axonal fibers. However, it is undetermined whether the downstream neurons are inhibited or excited by axonal stimulation. The present study addressed the question in rat hippocampus by in vivo experiments.

**Methods:**

Pulse stimulations with different frequencies (10–400 Hz) were applied to the Schaffer collateral, the afferent fiber of hippocampal CA1 region in anaesthetized rats. Single-unit spikes of interneurons and pyramidal cells in the downstream region of stimulation were recorded and evaluated.

**Results:**

Stimulations with a lower frequency (10 or 20 Hz) did not change the firing rates of interneurons but decreased the firing rates of pyramidal cells (the principal neurons) significantly. The phase-locked firing of interneurons during these stimulations might increase the efficacy of GABAergic inhibitions on the principal neurons. However, stimulations with a higher frequency (100–400 Hz) increased the firing rates of both types of the neurons significantly. In addition, the increases of interneurons’ firing were greater than the increases of pyramidal cells. Presumably, increase of direct excitation from afferent impulses together with failure of GABAergic inhibition might result in the increase of pyramidal cells’ firing by a higher stimulation frequency. Furthermore, silent periods appeared immediately following the cessation of stimulations, indicating a full control of the neuronal firing by the stimulation pulses during axonal stimulation. Furthermore longer silent periods were associated with higher stimulation frequencies.

**Conclusions:**

Low-frequency (10–20 Hz) and high-frequency (100–400 Hz) stimulations of afferent axonal fibers exerted opposite effects on principal neurons in rat hippocampus CA1. These results provide new information for advancing deep brain stimulation to treat different brain disorders.

## Background

Deep brain stimulation (DBS) with electrical pulses has been used for treating brain disorders such as Parkinson’s disease, dystonia and epilepsy [1–3]. In addition, DBS is promising for treating psychological diseases such as depression and obsessive–compulsive disorder [4, 5]. However, the mechanisms of DBS therapy remain unclear.

Previous reports have suggested that electrical pulses of DBS could inhibit neuronal firing because the effect of DBS is similar to surgical resections and because DBS can treat diseases caused by excessive excitations such as epilepsy [6]. A possible mechanism of the DBS-induced inhibition could be due to an increase of GABAergic inhibition by activating GABAergic afferents in the vicinity of stimulation electrodes [7, 8]. Besides the direct activation of GABAergic terminals, the stimulations might also induce inhibitions in the downstream neuronal networks through axonal projections.

Electrical stimulations can activate afferent axons, efferent axons and passing axons in the vicinity of the stimulation site. Because axons have a lower rheobase current and are more prone to be activated by electrical pulses of DBS than other neuronal elements [9], the pulse stimulations may activate axons even when the somata of neurons are inhibited [10]. The neuronal axons may then spread the stimulation-induced activation widely to downstream regions through projections of axonal fibers and may affect the activity of both excitatory and inhibitory neurons there [11].

Thresholds of action potential generation may be different for various types of neurons. For instance, in the hippocampus, interneurons (the inhibitory neurons) have a lower threshold than pyramidal cells (the principal neurons) [12]. Therefore, we hypothesized that the firing of pyramidal cells might be suppressed by the activation of local interneurons by certain types of stimulations at afferent fibers. Although studies of computational modeling have supported the hypothesis [13], it has not been validated by in vivo experiments. Both inhibitory effects and excitatory effects of stimulations on neurons have been reported by animal experiments [14, 15]. In addition, stimulations with various frequencies may change neuronal firing and result in different effects of DBS. Although a pulse frequency of 100–200 Hz is mostly utilized in current DBS applications for treating movement disorders [16], the effects of DBS at frequencies beyond this range may extend DBS applications to other diseases. It is unclear whether stimulations with different frequencies could selectively modulate the responses of inhibitory neurons and excitatory neurons.

To investigate whether axonal stimulations could activate neurons in the downstream networks selectively by changing stimulation frequency, we conducted in vivo experiments in anaesthetized rats by applying stimulations in the Schaffer collaterals, the afferent fibers of hippocampal CA1 region. A frequency range of 10–400 Hz was included in the study by extending the regularly used frequency range 100–200 Hz [16]. We analyzed the changes of single-unit activity of the two types of neurons (interneurons and pyramidal cells) in the downstream region of the stimulation site to evaluate the stimulation effects. The lamellar structures of axonal fibers, somata and dendrites in the hippocampal CA1 region provide separate and clear locations for the stimulation of afferent axons and for the recording of neuronal activity in the projecting region [17]. In addition, the hippocampus is one of the most important stimulation targets in DBS therapy for some brain diseases, such as refractory epilepsy [3, 18]. Therefore, the results of the present study could be significant for advancing the application of DBS.

## Materials and methods

### Animal experiments

The animal experiment was approved by the Institutional Animal Care and Use Committee, Zhejiang University. Seven adult Sprague–Dawley rats (male 250–350 g) were used in the study. The rats were anesthetized with urethane (1.25 g/kg, i.p.) and placed in a stereotaxic apparatus. Two stainless steel screws were fixed in the nose bone and were used as the reference and the ground of electrical signal recordings, respectively.

The recording electrode (a 16-channel microelectrode array, #Poly2, NeuroNexus Technologies, USA) was inserted into the hippocampal CA1 region (anteroposterior 3.5 mm, mediolateral 2.7 mm and dorsoventral ~ 2.5 mm to bregma) to obtain unit spikes of CA1 neurons. The stimulation electrode was a concentric bipolar stainless steel electrode with 250/75 μm diameters of outer/inner poles (100 μm length each) and 100 μm separation between the two poles (#CBCSG75, FHC Inc., USA). It was inserted (anteroposterior 2.2 mm, mediolateral 2.0 mm and dorsoventral ~ 2.8 mm to bregma) to stimulate the Schaffer collaterals to orthodromically activate the CA1 neurons at the recording site in the downstream of stimulation. The correct placements of the two electrodes were judged based on the waveforms of orthodromically evoked potentials serially in the recording array together with clear unit spikes appearing in the somatic layer [19].

### Recording and stimulation

The electrical signals collected by the recording array were first amplified by a 16-channel amplifier (Model 3600, A-M Systems Inc., USA) with a frequency band of 0.3–5000 Hz. Then, the signals were sampled at 20 kHz by a PowerLab data-acquisition system (PL 16/35, ADInstruments Inc., Australia).

**Fig. 1:**
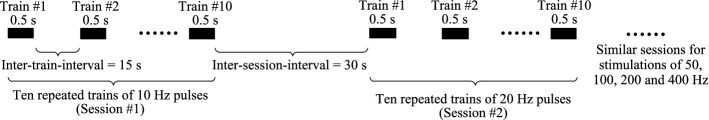
Timeline of the stimulation protocol

Trains of biphasic current pulses with a phase width of 0.1 ms were generated by a stimulator (Model 3800, A-M Systems Inc., USA). The pulse intensity was 20–35 μA. The duration of pulse train was 0.5 s. The pulse frequency was set as 10, 20, 50, 100, 200 and 400 Hz. To decrease stochastic disturbances, 10 trains with identical stimulation parameters were repeated with an inter-train-interval of 15 s within a stimulation session (see Fig. 1). Average values obtained from the 10 sweeps were used to evaluate the effects of stimulations with different frequencies. Pulse frequencies were monotonically increased across stimulation sessions [20].

### Data analysis

To obtain unit spikes, the stimulus artifacts in the raw signals were first removed by replacing the segments of stimulus artifacts with short interpolation lines [21]. Then, the signals were filtered by a digital high-pass filter with a cut-off frequency of 500 Hz to obtain the multiple unit activity (MUA). Spikes in MUA signals were detected by a threshold method, and single-unit spikes were obtained by spike sorting based on four recording channels close to the somatic layer. Finally, unit spikes from interneurons (Int) and pyramidal cells (Pyr) were distinguished based on spike waveforms and firing patterns. Details of the signal processing have been reported previously [14].

Two indexes were used to evaluate the neuronal responses to the stimulation trains: the firing rate of unit spikes and the length of silent period immediately following the cessation of stimulation. Dynamic curves of firing rates were calculated at a temporal resolution of 20 ms for a total period of 14.5 s, including 2 s before the onset of stimulation (baseline), 0.5 s during stimulation and 12 s after stimulation. Each data point was the mean value across 10 sweeps of stimulation trains with same parameters (see Fig. 1). In addition, the delay between each unit spike and its preceding pulse was used to describe the distribution of spikes in the inter-pulse-intervals.

Statistical data were represented as a mean with a standard deviation. Student *t* tests were used to evaluate the statistical differences of firing rates between baseline and stimulation period or between interneurons and pyramidal cells. One-way ANOVA with post-hoc Bonferroni multiple comparisons was used to evaluate the statistical differences among data groups with various stimulation frequencies.

## Results

### Modulating the firing of downstream neurons by orthodromic axonal stimulation

**Fig. 2:**
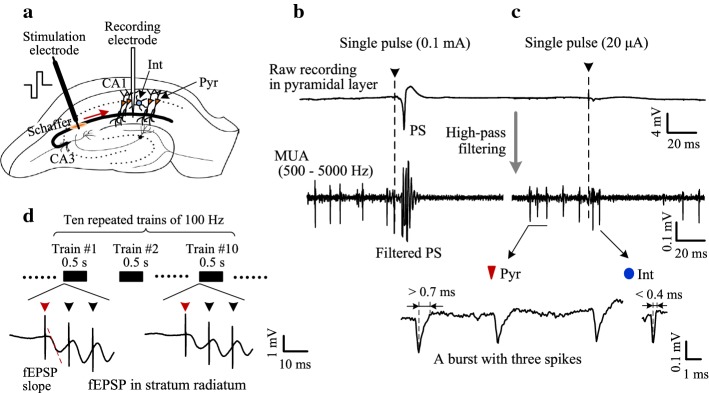
Action potentials evoked by the orthodromic stimulation of Schaffer collaterals in the hippocampal CA1 region. **a** Schematic diagram of the locations of recording electrode and orthodromic stimulation electrode. **b** Population spike (PS) evoked by a single pulse with a greater intensity 0.1 mA. **c** Unit spikes evoked by a single pulse with a smaller intensity 20 μA. Expanded waveforms below are examples of unit spikes from a pyramidal cell (Pyr) and an interneuron (Int), respectively. The baseline firing of the Pyr was a burst with three consecutive spikes. **d** Field EPSP (fEPSP) recorded in the stratum radiatum of CA1 region 200 μm from the pyramidal layer. Arrows denote stimulation artifacts with the red arrow indicating the first pulse of each stimulation train. The slope of fEPSP evoked by the first pulse was used to evaluate the strength of synaptic transmission

To obtain mere unit spikes in extracellular recordings during stimulation, we first determined the stimulation intensity. A single stimulation pulse applied to the Schaffer collaterals of hippocampal CA1 region could activate a bunch of axons to generate action potentials that would spread along the axons and agitate the downstream neurons (see Fig. 2a). If the intensity of the pulse was adequate (e.g., 0.1 mA), a population of downstream neurons would generate action potentials simultaneously. Because of the dense packing of the cell bodies in the hippocampal region [17], these evoked action potentials would superimpose together to form a potential waveform so-called population spike (PS) in the pyramidal layer. The PS would prevent the extraction of unit spikes immediately following stimulation pulses (Fig. 2b). Therefore, smaller intensities were tested until a single pulse only induced unit spikes without PS potentials (Fig. 2c). The weak intensity (e.g., 20 μA) was then used for the stimulation trains with various pulse frequencies (Fig. 1). In the stratum radiatum, the field EPSP (fEPSP) evoked by the first pulse of each stimulation train was distinguishable as long as the inter-pulse-interval was long enough, e.g. 10 ms for 100 Hz frequency (Fig. 2d).

**Fig. 3:**
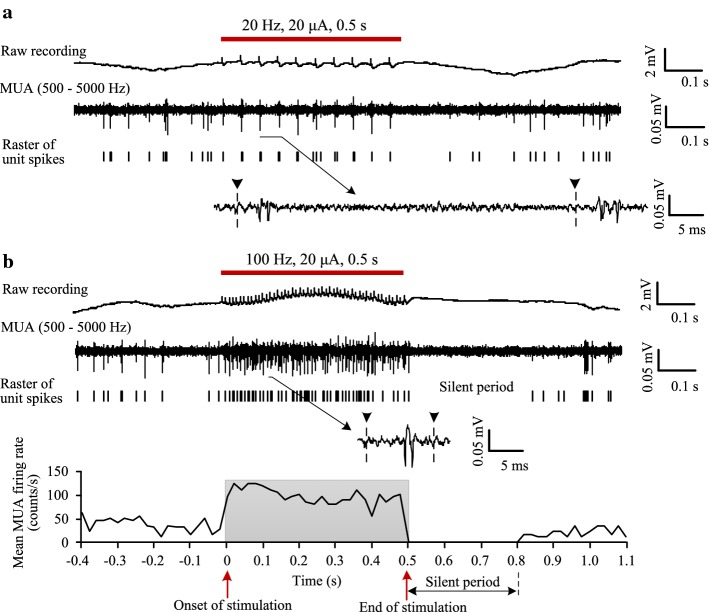
Modulating the neuronal firing in downstream region by stimulations of Schaffer collaterals. **a** An example of a pulse train (0.5 s) with a lower frequency of 20 Hz. Top to bottom: raw recording, MUA signal, raster plot of unit spikes, and a segment of expanded MUA signal. **b** Neuronal firing induced by pulse trains (0.5 s) with a higher frequency of 100 Hz. Top (4 rows): a typical sweep of stimulation. Bottom: dynamic changes of the mean firing rate of MUA calculated from 10 sweeps of identical stimulations. The red bars or the shadow area in plots denotes the stimulation period. The dashed lines with a “▼” in the expanded signals denote removed stimulation artifacts

With a lower stimulation frequency (e.g., 20 Hz), unit spikes always followed the pulses (Fig. 3a), indicating a strong control of the stimulation on the neuronal firing. However, the mean firing rate of MUA during the stimulation period (34.2 counts/s) was similar to the value (35.2 counts/s) in the baseline recording before stimulation. When the stimulation frequency was increased to 100 Hz (Fig. 3b), the mean firing rate of MUA during stimulation increased to 115 counts/s. In addition, a silent period (~ 0.3 s) without spikes appeared immediately following the cessation of stimulation (see the curve in the bottom of Fig. 3b).

These results indicated that stimulations of pulse trains could modulate the firing of downstream neurons and increase firing rates of neurons by a higher pulse frequency.

Because the stimulation of afferent axons (i.e., Schaffer collaterals) can simultaneously activate both interneurons and pyramidal cells in the downstream projection region (Fig. 2a), we next compared the firing of the two types of neurons.

### Frequency-dependent responses of the two types of neurons to stimulation trains

**Fig. 4:**
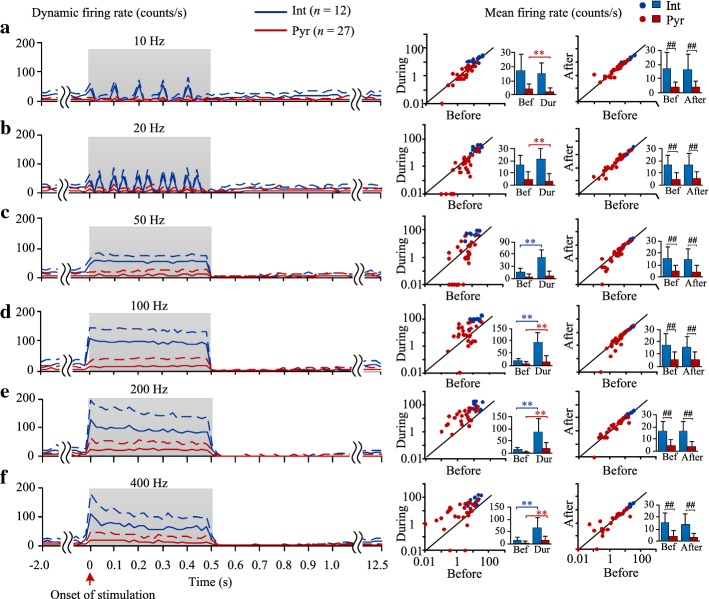
Changes of firing rates of the two types of neurons induced by stimulation trains (0.5 s) with various pulse frequencies. **a**–**f** Neuronal firing rates for stimulation frequencies of 10, 20, 50, 100, 200 and 400 Hz, respectively. Left: dynamic curves of the firing rates. The shadows denote the stimulation periods. The solid and dashed curves denote the means and standard deviations, respectively. Middle: comparisons of the mean firing rates between the periods before and during stimulation with scatter plots (in logarithmic coordinate axes) for each neuron and bar graphs for the two types of neurons. **P < 0.01, paired *t* tests between firing rates before and during stimulations. Right: comparisons of the mean firing rates between the periods before and after stimulation. ^##^*P* < 0.01, *t* tests between firing rates of interneurons and pyramidal cells. *n* = 12 for interneurons (Int) and *n* = 27 for pyramidal cells (Pyr)

To investigate the changes of neuronal responses induced by the 0.5-s stimulations, dynamic firing rates of individual interneurons (*n* = 12) and pyramidal cells (*n* = 27) were evaluated (Fig. 4a–f left). The mean firing rates in the three periods of before, during and after the stimulations with various pulse frequencies were also evaluated (Fig. 4a–f middle and right). For the period before stimulations, statistical analysis of one-way ANOVA showed no significant differences among the mean baseline firing rates with different frequencies for both interneurons (*F*_5,66_ = 0.07, *P* = 0.99) and pyramidal cells (*F*_5,156_ = 0.29, *P* = 0.91), indicating similar baseline neuronal states. Because the firing of unit spikes stopped for a short period immediately following the end of stimulation train (Fig. 3b), to evaluate the recovery of neuronal firing after stimulation, the post-stimulation firing rate was calculated in the time window of 10–12 s after the cessation of stimulation.

During stimulations with lower pulse frequencies (10 and 20 Hz), the interneurons generated a strong phase-locked firing following each pulse with a mean delay of 6.7 ± 5.9 ms (10 Hz) and 4.3 ± 2.1 ms (20 Hz) between unit spikes and the preceding pulse (Fig. 4a, b). The mean firing rates of interneurons (15.2 ± 7.2 and 21.3 ± 8.5 counts/s for 10 and 20 Hz, respectively) were not significantly different from the mean firing rates before stimulations (paired *t* test, *P* > 0.1, *n* = 12). However, the mean firing rates of pyramidal cells decreased significantly during stimulations (vs. the values before stimulations, paired *t* test, *P* < 0.01, *n* = 27). The mean delay of unit spikes of pyramidal cells was 40.8 ± 13.6 ms (10 Hz) and 19.1 ± 5.5 ms (20 Hz). The long delays with relatively large variances indicated that the firing of pyramidal cells was not phase-locked to the pulses of stimulation.

During stimulations with a higher pulse frequency of 50 Hz, the mean firing rates of interneurons increased significantly, whereas the mean firing rates of pyramidal cells did not change significantly (Fig. 4c).

During stimulations with further higher pulse frequencies (100, 200 and 400 Hz), the mean firing rates of both interneurons and pyramidal cells were significantly greater than the values before stimulation. In addition, during 200 and 400 Hz stimulations, the firing rates of interneurons showed a quick increase to a peak value near the onset of stimulation and then fell slightly to a steady-state (Fig. 4d–f).

**Fig. 5:**
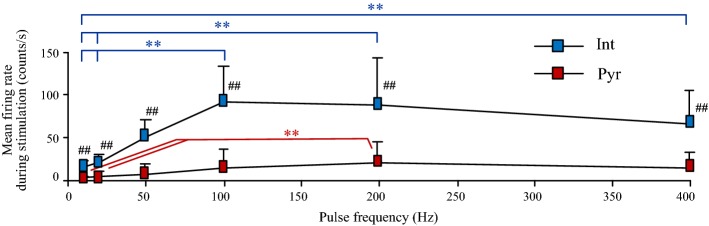
Comparisons of the neuronal firing rates among stimulations with various pulse frequencies and between two types of neurons during stimulations. **P < 0.01, post-hoc Bonferroni tests after significant ANOVA with *P* < 0.01. ^##^*P* < 0.01, *t* tests between interneurons and pyramidal cells

Interestingly, the firing rates of interneurons increased significantly with the increase of pulse frequency from 10 to 100 Hz and maintained steady at 100 and 200 Hz (93.2 ± 40.3 and 88.0 ± 54.9 counts/s, respectively) (Fig. 5). These firing rates at 100 and 200 Hz were significantly greater than the values at pulse frequencies of 10 and 20 Hz (15.2 ± 7.2 and 21.3 ± 8.5 counts/s; *P* < 0.01, post-hoc Bonferroni tests after significant ANOVA *P* < 0.01, *n* = 12). The firing rates of interneurons decreased slightly when the pulse frequency increased further to 400 Hz, but the value (65.9 ± 39.5 counts/s) was still significantly greater than the value at low frequency of 10 Hz (post hoc Bonferroni tests, *P* < 0.01, *n* = 12). During stimulations with a frequency over 50 Hz, the firing rates of all 12 interneurons increased (Fig. 4c–f middle, blue dots in the scatter plots).

The relative changes in firing rates of pyramidal cells with the increase of pulse frequency were similar to the changes of interneurons. During stimulations with a frequency below 20 Hz, more than 80% of the pyramidal cells decreased firing. During stimulations with a frequency of 100–400 Hz, 63–89% of the 27 pyramidal cells increased firing (Fig. 4a–f middle, red dots in the scatter plots). The firing rates of both types of neurons returned to baseline levels after stimulations (Fig. 4a–f right).

In addition, during stimulations with an identical pulse frequency, the mean firing rate of interneurons was always significantly greater than the value of pyramidal cells (4.3 to 8.8 multiples; Fig. 5; *t* test, *P* < 0.01). During baseline recordings before stimulation, the firing rates of interneurons were also significantly greater than the values of pyramidal cells (3.2 to 4.4 multiples; see Fig. 4a–f right; *t* test, *P* < 0.01). Taking the 100 Hz stimulation for example (Fig. 4d right), the firing rates of interneurons during baseline (17.2 ± 9.4 counts/s) and during stimulation (93.2 ± 40.3 counts/s) were all significantly greater than the corresponding values of pyramidal cells (6.1 ± 5.3 counts/s during baseline and 13.8 ± 16.9 counts/s during stimulation). Nevertheless, stimulations increased the difference between the firing rates of the two types of neurons from 3.2 multiples to 6.7 multiples.

To examine whether or not the successive short-stimulation trains with a higher frequency generated a long-term potentiation (LTP) in synaptic transmission, we took the first pulse of a train as a test pulse (Fig. 2d) to compare the slopes of fEPSP evoked by the first pulses of the first train and the last train (tenth). In the stimulation session of 100 Hz, the typical frequency to induce LTP, after nine preceding trains, the mean slope of fEPSP in the beginning of the tenth train (0.32 ± 0.31 mV/ms) was similar to that of the first train (0.31 ± 0.27 mV/ms; *n* = 7 rats, *P* > 0.1 paired *t* test). Similarly, in the stimulation session of 50 Hz, the mean slopes of fEPSP induced by the first pulses of trains were also similar (last train 0.30 ± 0.28 mV/ms vs. first train 0.32 ± 0.29 mV/ms; *n* = 7 rats, *P* > 0.1 paired *t* test). This indicated that the stimulation trains with a weak current intensity used in the present study did not induce obvious LTP.

These results indicated that during stimulations, the firing rates of interneurons and pyramidal cells increased in proportion to the pulse frequency till 100 Hz and then saturated even with the stimulation frequency reaching up to 400 Hz. In addition, the increases in firing rates of interneurons were greater than those of pyramidal cells. Compared to baseline values, the firing of pyramidal cells was suppressed by stimulations with lower frequencies but was enhanced by stimulations with higher frequencies. The firing of interneurons did not change significantly by stimulations with lower frequencies but was enhanced by stimulations with higher frequencies. The increase of neuronal firing by stimulation at a higher frequency was not caused by long-term changes in synaptic transmission.

### Neuronal activity following the cessation of stimulation

**Fig. 6:**
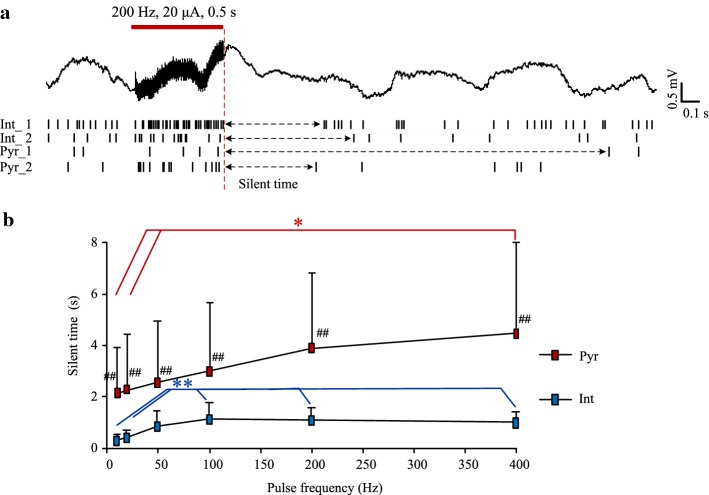
Silent periods of neuronal firing following 0.5-s stimulations with various pulse frequencies. **a** A recording example of 200 Hz stimulation. The red dashed line denotes the end of stimulation. The raster plots show the firing of 2 interneurons and 2 pyramidal cells. **b** The mean lengths of silent period of interneurons and pyramidal cells changed as a function of the pulse frequencies. *P < 0.05, post-hoc Bonferroni tests after ANOVA with *P* < 0.05. **P < 0.01, post-hoc Bonferroni tests after ANOVA with *P* < 0.01. ^##^*P* < 0.01, *t* tests between interneurons and pyramidal cells. *n* = 12 for interneurons (Int); *n* = 27 for pyramidal cells (Pyr)

Immediately following the cessation of stimulation, neurons stopped firing for a short period and then recovered gradually (Figs. 3b, 6a). Therefore, we next evaluated the post-effects of stimulation on the two types of neurons by the length of silent periods.

For the interneurons (Fig. 6b), the mean length of silent periods increased as the stimulation frequency increased from 10 to 100 Hz and then did not change significantly up to the frequency of 400 Hz. The mean lengths of silent periods with stimulations over 100 Hz (100 Hz: 1.1 ± 0.6 s; 200 Hz: 1.1 ± 0.4 s; 400 Hz: 1.0 ± 0.4 s) were all significantly longer than the values at lower frequencies (10 Hz: 0.3 ± 0.2 s; 20 Hz: 0.4 ± 0.2 s) (ANOVA *P* < 0.01; post-hoc Bonferroni tests, *P* < 0.01, *n* = 12).

For the pyramidal cells (Fig. 6b), the mean length of silent periods increased as the stimulation frequency increased from 10 to 400 Hz. However, only the silent period at 400 Hz (4.2 ± 3.5 s) was significantly longer than the silent periods at lower frequencies (10 Hz: 1.9 ± 1.8 s; 20 Hz: 2.3 ± 2.4 s) (ANOVA, *P* < 0.05; post-hoc Bonferroni tests, *P* < 0.05, *n* = 27). The lack of significant differences among other groups might be caused by the great variances of silent periods of pyramidal cells. In addition, the mean duration of silent periods of pyramidal cells (> 2 s) was always significantly longer than the mean value of interneurons (≤ 1.1 s, Fig. 6b).

These results showed that the silent period of interneurons increased significantly with stimulation frequencies over 100 Hz, which was parallel to the changes of neuronal firing rates during stimulations (Fig. 5).

## Discussion

The major findings of the present study are as follows. (1) During axonal stimulations with lower frequencies (10 and 20 Hz), the firing rates of interneurons did not change significantly, whereas the firing rates of principal neurons (the pyramidal cells) decreased significantly. (2) During stimulations with higher frequencies (100–400 Hz), the firing rates of both types of neurons increased significantly. In addition, the increases of interneurons’ firing rates were greater than the increases of pyramidal cells. (3) Longer silent periods appeared immediately following stimulations with higher frequencies that induced more neuronal firing during stimulations. Possible underlying mechanisms and implications of these findings are analyzed below.

First, the decrease of pyramidal cells’ firing by stimulations of lower frequencies could be caused by the inhibition from the GABAergic interneurons. Although the mean firing rates of interneurons did not increase significantly during the stimulations with lower frequencies, their phase-locked firing could increase the synchronization of firing among different interneurons thereby increasing the efficacy of GABAergic inhibitions on the principal neurons (the pyramidal cells, see Fig. 4a, b). Stimulation pulses applied to the Schaffer collaterals, i.e., the afferent fibers of hippocampal CA1 region, can simultaneously activate both interneurons and pyramidal cells in the downstream region through mono-synaptic connections (Fig. 2a) [17]. However, with the weak stimulation intensities used in the study, the interneurons might be activated with higher fidelity than the pyramidal cells by the pulses of lower frequencies (10 and 20 Hz) because of the lower thresholds of action potential generation for interneurons [12]. The short mean delay of interneurons’ spikes indicated that the interneurons were activated directly by the pulses; whereas the pyramidal cells were not activated directly by the pulses because of the long and variable delays of their spikes. Presumably, the activated interneurons may suppress the pyramidal cells through the effect of feed-forward inhibition thereby decreasing the firing of pyramidal cells [17, 22].

Second, the increase of stimulation frequency may change the inhibitory effect of stimulation on pyramidal cells into an excitatory effect. Stimulations with higher frequencies (100–400 Hz) significantly increased the firing rates of both types of neurons (Fig. 4). The increases of neuronal firing may be caused by the increase of afferent impulses from the high-frequency stimulations (HFS). Nevertheless, the significant increase of pyramidal cells’ firing indicated that the increase of GABAergic inhibitions from interneurons failed to counteract the increase of excitatory effects from afferent impulses, although the increased firing rates of interneurons were 5–6 multiples of the firing rates of pyramidal cells. One possible explanation of the result may be that a higher rate of interneurons’ firing does not necessarily result in a corresponding increase in GABAergic inhibitions on pyramidal cells. With repeated activation of interneurons by HFS, GABAergic synapses at the terminals of interneuron’s axons may be impaired and may even transfer from inhibitory to excitatory effects [23, 24]. The change of GABAergic synapses could limit the increase of inhibitory effects of interneurons on the pyramidal cells. Therefore, the increase of direct excitation from afferent impulses together with the failure of GABAergic inhibition might result in the increase of pyramidal cells’ firing during stimulations with higher frequencies.

Increase of neuronal firing by HFS could also be caused by changes of synaptic plasticity, such as LTP. However, based on the requirement of cooperativity, an intensity threshold must be reached to activate substantial afferent fibers to induce LTP [25]. Therefore, the stimulations with weak current intensities used in the present study did not induce substantial LTP, as indicated by the consistent fEPSP after trains of stimulation with the typical frequency 100 Hz for LTP generation. Nevertheless, synaptic plasticity-related changes induced by HFS around 100 Hz may contribute to the effects of DBS [16]. Utilizing neural plasticity might be a new direction of DBS development to treat certain disorders which still needs further investigations.

Third, in contrast to the increased neuronal firing during HFS over 100 Hz, a prolonged silent period with no neuronal firing immediately followed the cessation of HFS (Figs. 4d–f, 6). This silent period indicates that the neuronal firing during HFS was induced by stimulation, not by neuronal activity transmitting from upstream regions. HFS-induced axonal block may prevent the upstream neuronal activity and cause the silent periods [26, 27]. Nevertheless, a portion of the stimulation excitation may still act on the downstream neurons through partial blockade of axons [14]. In addition, the HFS-induced axonal failure may also explain why the neuronal firing did not increase further with the increase of pulse frequency over 100 Hz (Fig. 5). Because the HFS-induced axonal block is frequency dependent [27, 28], pulses of a higher frequency that generate more but weaker excitation may result in a similar amount of net excitation on the downstream neurons as the pulses of a lower frequency generate less but greater excitation. Therefore, the neuronal firing was limited during HFS with frequencies over 100 Hz.

Finally, the previous studies on pallidal stimulation also showed that weak-intensity stimulations can inhibit neuronal firing by activating GABAergic axon terminals [15, 29]. However, in those studies, the GABAergic inhibition was induced in the close vicinity of stimulation electrode and inhibited pallidal neurons at the stimulation site, not in the downstream projection regions. Studies have shown that pulse stimulations can generate action potentials in efferent axons even though the firing of neuronal cell bodies is suppressed at stimulation sites [10]. The action potentials traveling along the efferent axons to the downstream neurons may play an important role in DBS therapy [30, 31]. The present study demonstrates the changes of dynamic balance between inhibition and excitation in the downstream neurons induced by axonal stimulations, thereby providing new insights into different effects of various stimulations. In addition, the results of weak current stimulation are significant not only for DBS but also for transcranial neuronal stimulation [13, 32].

## Conclusion

Stimulations of afferent axons with a low frequency (e.g., 10, 20 Hz) may inhibit the firing of principle neurons by activating interneurons in the downstream projection regions. However, the inhibitory effect of interneurons may be overwhelmed by the excitatory impulse of stimulations with a higher frequency over 100 Hz, resulting in a net effect of excitation but not inhibition on the principle neurons. The opposite effects induced by axonal stimulations with different frequencies provide new clues for developing various paradigms of deep brain stimulation to treat different brain disorders.

## Data Availability

All data generated or analyzed during this study are included in this published article.

## Abbreviations

DBS: deep brain stimulation
HFS: high-frequency stimulation
PS: population spike
Pyr: pyramidal cell
Int: interneuron
LTP: long-term potentiation
